# ASO Author Reflections: Challenges of Circulating Tumor DNA in the Management of Gastrointestinal Peritoneal Carcinomatosis

**DOI:** 10.1245/s10434-022-12543-8

**Published:** 2022-09-13

**Authors:** Brittany G. Sullivan, Farshid Dayyani, Maheswari Senthil

**Affiliations:** 1 Division of Surgical Oncology, Department of Surgery, University of California Irvine, Orange, CA USA; 2 Division of Hematology-Medical Oncology, Department of Medicine, University of California Irvine, Orange, CA USA

The widespread adoption of circulating tumor DNA (ctDNA) liquid biopsy in oncology clinical practice has resulted in major shifts in treatment paradigms.^1^ Several ongoing clinical studies have been designed to expand the scope of ctDNA-informed treatment decisions in gastrointestinal (GI) cancers.^1,2^

Although expansion of ctDNA-based treatment decisions is a necessary advancement in cancer care, it is crucial to understand the limitations of this test and thereby the clinical situations in which ctDNA liquid biopsy may not be entirely reliable. A growing body of evidence shows the interaction of tumor type, tumor burden, and site of metastases on the amount of ctDNA detected in the blood. Specifically, ctDNA is often undetected in primary mucinous GI tumors, a histology frequently associated with peritoneal carcinomatosis.^3^ In addition, studies exclusively consisting of patients with either gastric or colon cancer have shown that ctDNA is either undetected or detected at extremely low levels in patients with peritoneal carcinomatosis (PC).^4,5^ Since  PC is a common form of metastasis in GI malignancies and certain aspects of PC pathophysiology are shared across the different GI primary cancers, we evaluated the phenomenon that PC of GI origin may be associated with lower ctDNA levels.

Our study of 279 patients with stages 2–4 GI malignancies found that the amount of plasma ctDNA is significantly lower in GI malignancies with PC than in visceral metastases.^6^ The significance of this observation is further enhanced by the fact that the majority of the patients in this study had large volume of peritoneal disease burden, with an average of six intrabdominal regions involved. We also found that plasma ctDNA next-generation sequencing (NGS) showed fewer genomic alterations in patients with GI PC than tissue analysis, with a concordance rate of merely 18%.^6^

These observations underscore the important biologic differences in the shedding and transport of ctDNA between PC and visceral metastasis. Detection of ctDNA is affected by the anatomy of the peritoneum and peritoneal fluid transport, as well as by the histology of tumors that have a predilection to metastasize to the peritoneum. Specifically, the physical separation of the peritoneal cavity and systemic circulation by the blood-peritoneal barrier and the poor vascularization of peritoneal metastasis result in decreased access of ctDNA to systemic circulation.^7^

In patients with PC, the common indications for ctDNA testing to detect actionable genomic alterations and the evaluation of treatment responses should be used and interpreted with caution. Although, molecular residual disease was beyond the scope of our study, it is reasonable to extrapolate, both based on the findings from our study and others, that a negative ctDNA testing to detect molecular residual disease in tumors with increased predilection for PC should be interpreted with high-degree of suspicion because they may represent a false-negative result. A recent study of a tumor-informed assay (Signatera^TM^, Natera, Austin, TX) published by our group showed that ctDNA was detected in only 53% of the patients with PC.^8^

Although advances to improve the sensitivity of ctDNA detection such as DNA methylation markers may improve detection, the fundamental anatomic and pathologic differences in PC will continue to be a challenge. These challenges offer a rich opportunity to explore other liquid biopsy approaches, such as exosome-based liquid biopsy, that may be more reliable than ctDNA in PC.^9^ The role of exosomes in cancer growth and metastasis is now well-recognized due to the growing body of evidence demonstrating the effects of exosomes in creating a premetastatic niche and cancer progression. The stability of exosomes and the rich information carried by these nanovesicles make them an ideal target for liquid biopsy. An initial study by our group has shown that the plasma exosome-gene signature is able to detect PC 100% of the time (unpublished). Further validation studies in prospective cohort of patients are currently underway to confirm the results of our initial observation. Hence, simultaneous efforts to improve the sensitivity of ctDNA together with deep exploration of alternate, more sophisticated liquid biopsies such as exosomes will help continue to move the field forward with the ultimate goal of assisting physicians make customized, risk-adjusted treatment decisions with confidence.
